# Tissue miRNA 483-3p expression predicts tumor recurrence after surgical resection in histologically advanced hepatocellular carcinomas

**DOI:** 10.18632/oncotarget.24860

**Published:** 2018-04-03

**Authors:** Francesco Vasuri, Silvia Fittipaldi, Vanessa De Pace, Laura Gramantieri, Valentina Bertuzzo, Matteo Cescon, Antonio D. Pinna, Michelangelo Fiorentino, Antonia D’Errico, Matteo Ravaioli

**Affiliations:** ^1^ Institute of Oncology and Transplant Pathology, Department of Experimental Diagnostic and Specialty Medicine, S.Orsola-Malpighi Hospital, Bologna University, Bologna, Italy; ^2^ Unit of General Surgery and Transplantation, Department of Medical and Surgical Sciences, S.Orsola-Malpighi Hospital, Bologna University, Bologna, Italy; ^3^ Internal Medicine, Department of Medical and Surgical Sciences, S.Orsola-Malpighi Hospital, Bologna University, Bologna, Italy

**Keywords:** hepatocellular carcinoma, IGF2, liver resection, miRNA, tumor recurrence

## Abstract

The choice of surgical treatment for hepatocellular carcinoma (HCC) depends on several prognostic variables, among which histological features, like microvascular invasion and tumor grade, are well established. This study aims to identify the tissue miRNAs predictive of recurrence after liver resection in “histologically advanced” HCC. We selected 54 patients: 15 retrospective resected patients without recurrence (group A), 19 retrospective resected patients with HCC recurrence (group B), and 20 prospective patients (group C), with 4 recurrence cases. All selected HCC were “histologically advanced” (high Edmondson grade and/or presence of microvascular invasion). A wide spectrum of miRNAs was studied with TaqMan Human microRNA Arrays; qRT-PCR assays were used to validate results on selected miRNAs; immunohistochemistry for IGF2 was applied to study the mechanism of miR-483-3p. As a result, a significant differential expression between group A and B was found for 255 miRNAs. Among them we selected miR-483-3p and miR-548e (P<0.001). As a single variable (group C), HCC with miR-483-3p downregulation (mean fold increase 0.21) had 44.4% of recurrence cases; HCC with miR-483-3p upregulation (mean fold increase 5.94) showed no recurrence cases (P=0.011). At immunohistochemistry (group C), the HCC with loss of cytoplasmic IGF2 expression showed a down-regulation of miR-483-3p (fold increase 0.57). In conclusion, in patients with “histologically advanced” HCC, the analysis of specific tissue miRNAs (particularly miR-483-3p) could help identify the recurrence risk and choose which treatment algorithm to implement (follow-up, resection or transplantation). This could have an important impact on patient survival and transplantation outcome, improving organ allocation.

## INTRODUCTION

Hepatocellular carcinoma (HCC) is one of the most aggressive malignancies and the fifth leading cause of cancer-related death worldwide [1]. HCC is characterized by a poor prognosis, due to high recurrence rates and limited treatment choices [2, 3]. Surgical treatments for HCC, liver resection or liver transplantation, are chosen according to the radiological stage, the occurrence of cirrhosis and the liver function [4]. Liver resection for intermediate or advanced HCC (multiple lesions and/or microvascular invasion) shows 25% of recurrence-free survival at 5 years [5], *versus* 90% with liver transplantation [6].

Histological features of HCC, like microvascular invasion and tumor grade are well-known prognostic variables, but they are not always available pre-operatively [7–12]. Recently, many studies have addressed this problem, trying to identify molecular profiles of advanced HCC. The availability of markers predictive of local recurrence and/or metastatic disease might significantly improve the management and survival of HCC patients when surgical procedures, resection and transplantation, are adequately applied.

MicroRNAs (miRNAs) are non-coding small RNA (20-22 nucleotides) that potentially control the expression of at least 20-30% of all human transcripts and, therefore, they are likely to be involved in almost all basic signaling pathways [13]. miRNAs contribute to a variety of pathological events, including several types of human cancer [14–16]. Thus, miRNAs are able to inhibit the expression of the major tumor-related genes in carcinogenesis, acting themselves as oncogenes or tumor suppressors [17, 18].

Many miRNAs are implicated in the development of HCC [19], mainly in cell proliferation, resistance to programmed cell death, evasion of growth suppression, cell immortalization, angiogenesis, as well as invasion and metastatization abilities. miRNAs are also involved in the inflammatory environment, as in cases of chronic hepatitis and fibrosis [20]. Finally, miRNAs can also be implicated in genomic instability and hepatocyte metabolic pathways, which seem to be related to tumorigenesis [21]. Notwithstanding, the implementation of miRNAs in the routine clinical management of HCC patients has made very little progress.

The present study aims to identify the tissue miRNAs predictive of recurrence after liver resection from a selected series of HCC with histological features of tumor aggressiveness.

## RESULTS

### Patients and histopathological features

**Figure 1:**
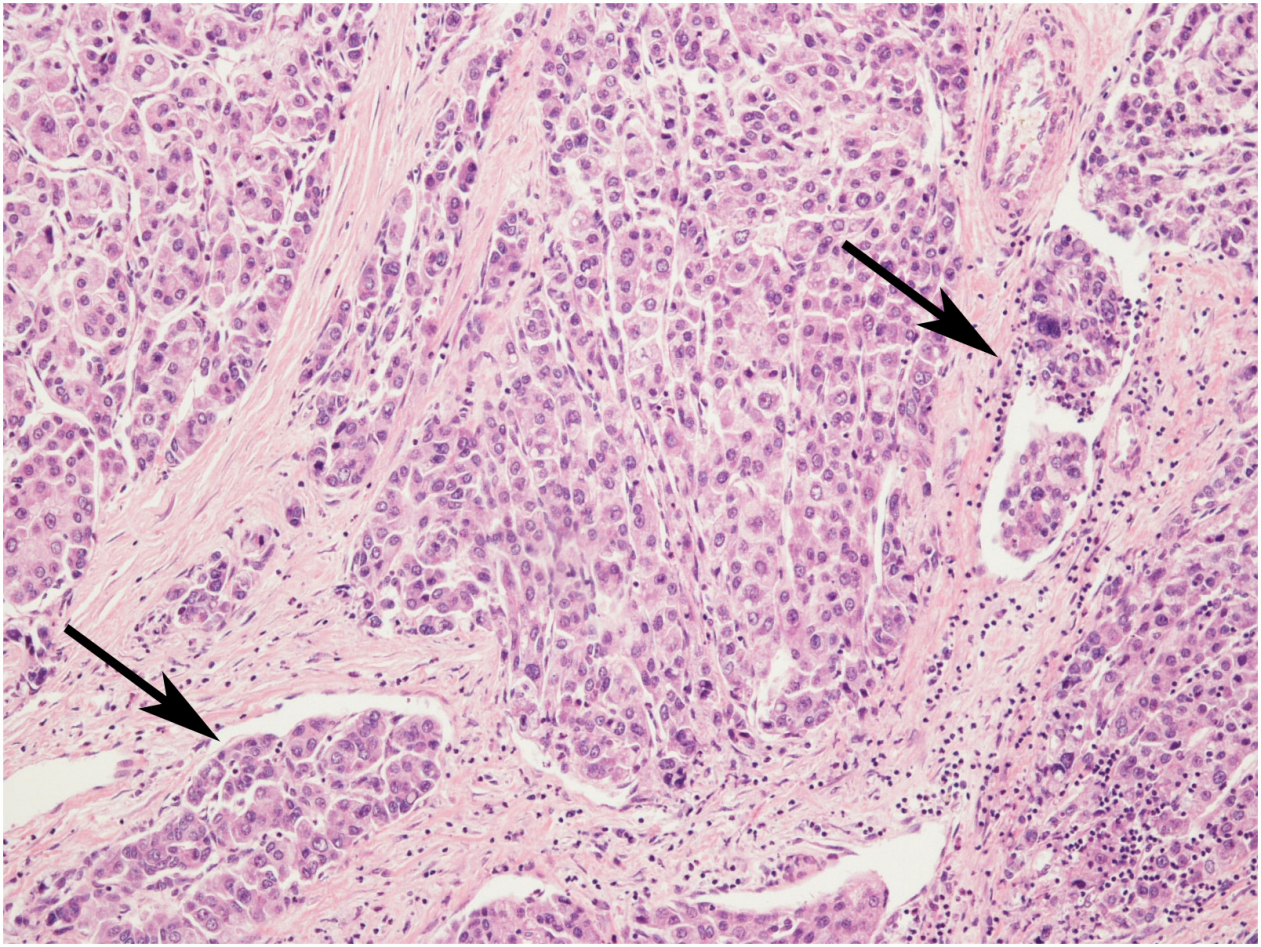
An example of “histologically advanced” hepatocellular carcinoma, characterized by Edmondson’s grade 3 and several microvascular invasions (arrows) Hematoxylin-Eosin stain, magnification 20x.

We selected 54 HCC patients, sorted in 3 groups (see Materials and Methods). All HCC were “histologically advanced”, i.e. with Edmondson grade 3 or 4, and/or presence of microvascular invasion. Since we selected “histologically advanced” HCC, we obtained a high incidence of unfavorable histological variables (Figure 1, Table 1): Edmondson grade 3 or 4 was present in 49 out of 54 (90.7%) cases, microvascular invasion in 47 (87.0%), infiltrative margins in 40 (74.1%), and at least one area of solid architecture was present in 30 (55.6%). Mean tumor dimensions were 5.76±4.60 cm (range 1.20-23.00 cm). In particular, patients in retrospective groups A and B represent a homogeneous population for clinical and pathological characteristics. No significant differences were observed between group A and group B features that are presented in Tables 1 and 2 (data not shown), except for the presence of solid architecture, which was recorded in 46.7% of group A and 89.5% of group B (p=0.009).

**Table 1:** Cancer-related characteristics of the study population

	*Retrospective cohort*		*Prospective cohort*	
	Group A	Group B	Group C	Total
**Patients (N)**	15	19	20	54
**Tumor**				
*Single, N (%)*	12 (80)	13 (68.4)	9 (45)	34 (63)
*Multiple, N (%)*	3 (20)	6 (31.6)	11 (55)	20 (37)
*AFP (ng/mL)*				
<30	10 (66.7)	14 (73.7)	12 (60)	36
>30	5 (33.3)	5 (26.3)	8 (40)	18
**Local ablative therapies before surgery**				
**Patients (N)**	4	5	10	19
*PEI, N (%)*	1 (6,6)	1 (5.3)	1 (5)	3 (5.5)
*RF, N (%)*	2 (13.3)	0	5 (25)	7 (13)
*TACE, N (%)*	2(13.3)	4 (21)	7 (35)	13 (24)
*TARE, N (%)*	0	0	2 (10)	2 (3.7)
**Histopathological features**				
Edmondson's grade 3/4, N (%)	12 (80)	18 (94.7)	19 (95)	49 (90.7)
Microvascular Invasion, N (%)	12 (80)	17 (89.5)	18 (90)	47 (87)
Infiltrative margins, N (%)	13 (86.7)	16 (84.2)	11 (55)	40 (74.1)
Solid architecture, N (%)	7 (46.7)	17 (89.5)	6 (30%)	30 (55.6)

AFP, alpha-fetoprotein; PEI, percutaneous ethanol injection; RF, radiofrequency ablation; TACE, transarterial chemoembolization; TARE, transarterial radioembolization.

**Table 2:** Baseline clinical characteristics of the study population

	*Retrospective cohort*		*Prospective cohort*	
	Group A	Group B	Group C	Total
**Patients (N)**	15	19	20	54
**Sex**				
*Male, N (%)*	11 (73.4)	17 (89.5)	17 (85)	45 (83.3)
*Female, N (%)*	4 (26.6)	2 (10.5)	3 (15)	9 (16.7)
**Age**				
<50, N (%)	4 (26.6)	1 (5.3)	3 (15)	8 (14.8)
>50, N (%)	11 (73.4)	18 (94.7)	17 (85)	46 (85.2)
**BMI**				
<30, N (%)	14 (93.4)	15 (79)	17 (85)	46 (85.2)
>30, N (%)	1 (6.6)	4 (21)	3 (15)	8 (14.8)
**Cirrhotic patients, N (%)**	12 (80)	13 (68.4)	15 (75)	40 (74)
**Etiology of liver disease**				
*HCV, N (%)*	9 (60)	8 (42.1)	8 (40)	25 (46.3)
*HBV, N (%)*	3 (20)	5 (26.3)	6 (30)	14 (25.9)
*Metabolic, N (%)*	1 (6.6)	1 (5.3)	1 (5)	3 (5.6)
*Alcoholic, N (%)*	1 (6.6)	3 (15.7)	0	4 (7.4)
*Biliary, N (%)*	0	0	2 (10)	2 (3.7)
*Cryptogenic, N (%)*	1 (6.6)	1 (5.3)	0	2 (3.7)
*None, N (%)*	0	1 (5.3)	3 (15)	4 (7.4)
**Pre-operative liver Function**				
*AST*				
<50, N (%)	6 (40)	13 (68.4)	7 (35)	26 (48.1)
>50, N (%)	9 (60)	6 (31.6)	13 (65)	28 (51.9)
*ALT*				
<50, N (%)	6 (40)	12 (63.1)	10 (50)	28 (51.9)
>50, N (%)	9 (60)	7 (36.9)	10 (50)	26 (48.1)
*ALP*				
<120, N (%)	5 (33.3)	7 (36.9)	12 (60)	24 (44.4)
>120, N (%)	10 (66.7)	12 (63.1)	8 (40)	30 (5.6)
*MELD*				
<9, N (%)	11 (73.3)	16 (84.2)	9 (45)	36 (66.7)
10-19, N (%)	4 (26.7)	2 (10.5)	6 (30)	12 (22.2)
>20, N (%)	0 (0)	1 (5.3)	5 (25)	6 (11.1)

ALP, alkaline phosphatase; ALT, alanine transaminase; AST: aspartate transaminase; BMI, body-mass index; MELD, model for end-stage liver disease.

### Differential miRNA expression in recurrent and non-recurrent HCC: explorative phase

**Figure 2:**
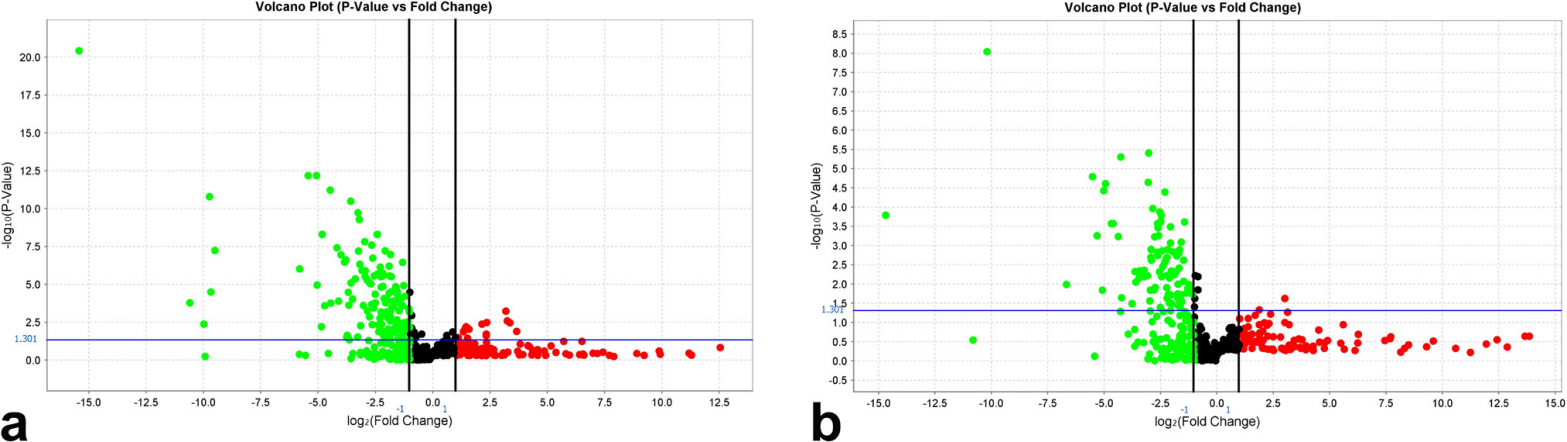
Volcano plots representing miRNAs deregulated in HCC according to the normalization in **(a)** recurrent and **(b)** non-recurrent HCC. The X axis represents the log2 (fold change) and the Y axis the -log10 (P value). miRNAs with statistically significant differential expression (P<0.05 adjusted for multiple testing) are located above the horizontal threshold line and outside the pair of vertical threshold black lines. Down-regulated miRNAs are found in the upper left (green) of the plot and up-regulated miRNAs in the upper right parts (red).

A total of 255 miRNAs showed a significant differential expression according to the presence (7 cases from group B, Figure 2a) or absence (13 cases from group A, Figure 2b) of tumor recurrence after resection. In particular, 101 miRNAs were deregulated (99 down-regulated) in non-recurrent HCC, and 154 miRNAs were deregulated (134 down-regulated) in recurrent HCC (Supplementary Table 1).

### Differential miRNA expression in recurrent and non-recurrent HCC: validation phase

Based on the statistical analysis performed on the arrays with the ExpressionSuite Software, we selected two promising miRNAs for the validation phase: miR-483-3p and miR-548(e) (also named miR-548e-3p). Both were deregulated in the recurrence group compared to the non-recurrent HCC group. The student’s t test showed that the differences in mean levels of expression in the 2 groups in arrays were highly significant; for 483-3p, p=0.0000001 and for 548e-3p = 0.0009482. As mentioned above, the t-test compared the means of the normalized Ct values between the two groups and the p value were adjusted with the Benjamini–Hochberg method to control the type I error rate. Moreover, from a review of the literature, we saw that both miR-483-3p and miR-548e have a broad range of targets (respectively 377 targets and 934 targets, miRDB - miRSearch V3.0), although there is missing evidence in the literature concerning their role in human cancers, especially for miR-548e. miR-483-3p is known to be deregulated in different tumors including Wilms' tumors, colon, breast and liver cancers [22]. We therefore decided to validate the expression of these two miRNAs on the whole series (54 patients) by performing single PCR assays to quantify miR-483-3p and miR-548(e) levels.

Single PCR assays confirmed the significant differential expression of miR-483-3p (fold increase 8.18±3.04; p=0.012, Wilcoxon-matched pairs test) and miR-548(e) (fold increase 9.17±39.66; p<0.001) in HCC tissue compared to controls.

miR-483-3p was significantly down-regulated in recurrent HCC (22 cases) compared to non-recurrent HCC (32 cases), with mean fold-increase values of 0.53±0.91 and 15.04±54.06 respectively (p=0.025, Wilcoxon-matched pairs test). miR-548(e) was also significantly down-regulated in recurrent HCC, which showed a mean fold-increase of 0.48±0.92 compared to a mean fold-increase of 16.99±53.85 in non-recurrent HCC (p=0.002).

Of note, no differences were found in miRNA expression between transplanted and resected HCC from group C: the mean fold increase of miR-483-3p was 6.87±15.36 and 4.63±11.73 in transplanted and resected HCC respectively (p=0.065); the mean fold increase of miR-548(e) was 9.91±27.80 and 38.83±85.99 in transplanted and resected HCC respectively (p=0.165).

### Survival analysis

**Figure 3:**
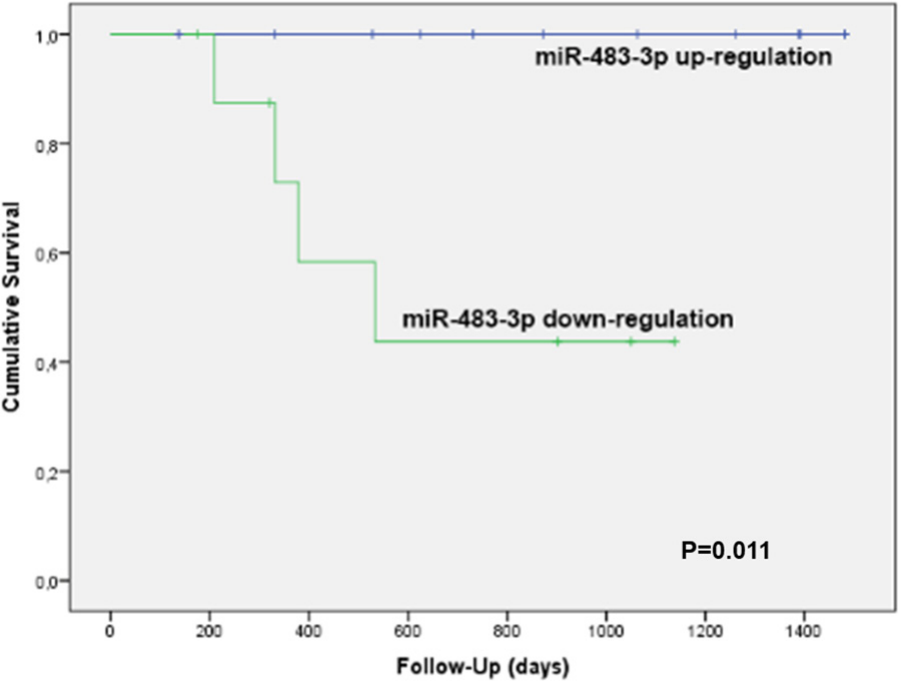
Kaplan-Meier’s recurrence-free survival curve of the 20 prospective patients of group C according to the expression of miR-483-3p The blue line (no recurrence cases) refers to the 11 cases with a mean miR-483-3p fold increase of 5.94 (miR-483-3p up-regulation), while the green line (4 recurrence cases) refers to the 9 cases with a mean miR-483-3p fold increase of 0.21 (down-regulation). This survival curve is statistically significant (Log Rank P=0.011).

In order to study the significance of miR-483-3p and miR-548e expression in recurrence-free survival of the 20 prospectively enrolled patients of group C, we elaborated an ROC curve to find a cut-off value. For miR-483-3p the area under the curve (AUC) was 0.771, and with a ΔΔCT value of -0.075we found an 80% sensitivity and 64% specificity towards HCC recurrence. The survival curve obtained with this cut-off was statistically significant (Log Rank P=0.011, Figure 3). In particular, no cases of local HCC recurrence were observed with a miR-483-3p ΔΔCT above the cut-off: this was observed in 11/20 cases with a mean miR-483-3p ΔΔCT value of -2.57±2.66, corresponding to a mean fold increase of 5.94 of miR-483-3p expression. Conversely, a miR-483-3p ΔΔCT below the cut-off was observed in 9/20 cases, with 4 local HCC recurrences (44.4%); in this group the mean miR-483-3p ΔΔCT value was 2.27±1.56, corresponding to a mean fold increase of 0.21. This means that a down-regulation of miR-483-3p implies a risk of tumor recurrence, while an up-regulation seems to be a protective factor, and it seems to be applicable in both resected and transplanted cases.

The recurrence-free survival curve built with miR-548e ΔΔCT did not reach statistical significance (data not shown).

### Expression of miR-483-3p-related proteins: immunohistochemistry for IGF2

In the attempt to shed light on the mechanism behind the prognostic impact of miR-483-3p, we performed IHC for IGF2, an important growth factor co-expressed with miR-483-3p [22], on the 20 prospective HCC from Group C.

**Figure 4:**
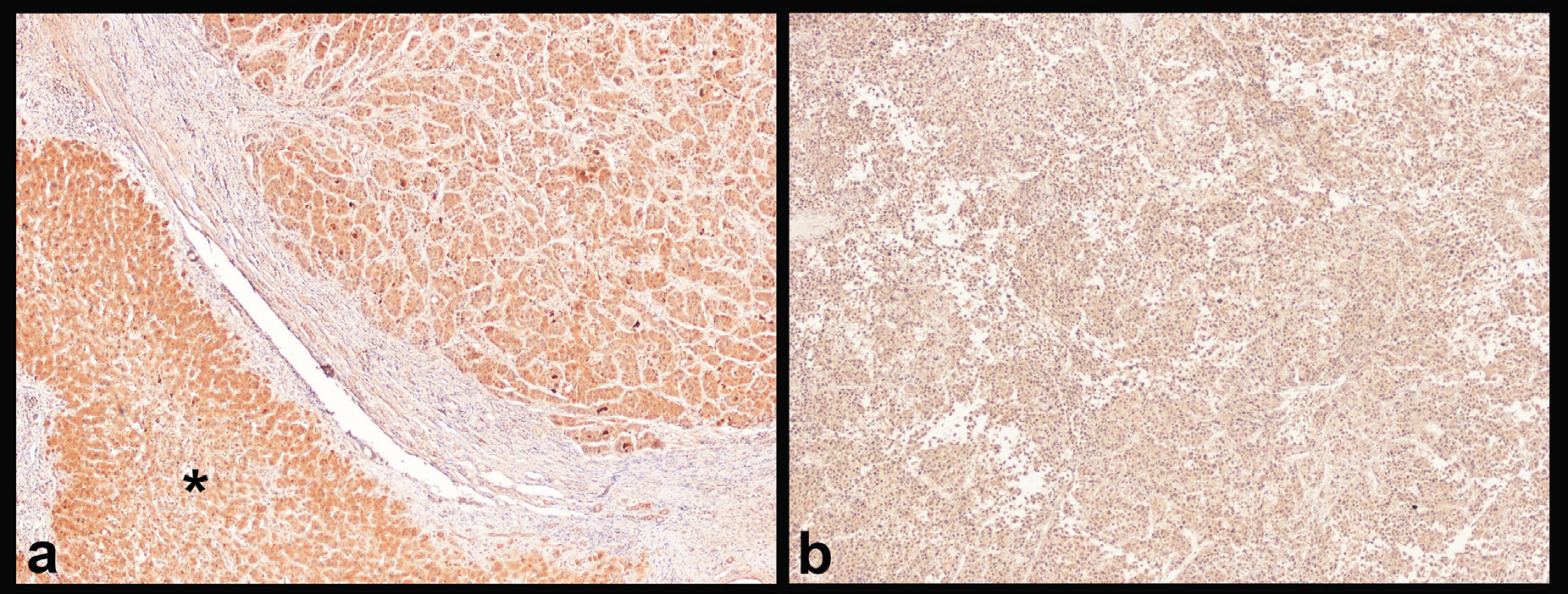
Immunohistochemistry for IGF2 **(a)** HCC with strong cytoplasmic IGF2 positivity on both tumoral tissue (upper right) and non-neoplastic liver (asterisk). **(b)** HCC with loss of IGF2 cytoplasmic positivity; this loss of positivity seems to be connected to the down-regulation of miR-483-3p. Magnification 4x.

We found a loss of cytoplasmic positivity for IGF2 in 6 (30%) HCC, while the IHC expression was preserved (i.e. of the same intensity as in non-neoplastic liver) in the remaining cases (Figure 4). Interestingly, the mean miR-483-3p ΔΔCT of the 6 cases with loss of tissue IGF2 was 0.82±2.14, corresponding to a 0.57 fold increase (down-regulation). The mean miR-483-3p ΔΔCT of the 14 cases with preserved expression of tissue IGF2 was -0.71±2.92, corresponding to a 1.62 fold increase (up-regulation). This difference does not reach statistical significance (P=0.274, ANOVA), probably due to the small sample size, but the co-expression of miR-483-3p and IGF2 is likely to be maintained also in our study.

## DISCUSSION

HCC is a widespread malignant neoplasm still burdened by a poor prognosis, due to a high recurrence rate after surgery and the lack of a standardized adjuvant therapy [1–3]. The histopathological HCC features, including tumor grade, growth pattern, architecture, and occurrence of MVI still represent the most important predictors of tumor recurrence [7–12]. The biological behavior of HCC is a determining factor in planning the surgical treatment, i.e. liver resection or liver transplantation: these two treatment strategies are applied alternatively or sequentially according to the tumor stage and liver function. When feasible, the principal limitation of liver resection remains the high rate of tumor recurrence, and some authors suggest planning a liver transplantation even before any recurrence in case unfavorable biological features are discovered after resection, especially micro-vascular invasion or/and high tumor grade [23]. This strategy is meant to prevent the risk of finding that the patient is not suitable for liver transplantation due to tumor recurrence outside the criteria of transplantability [11]. On the other hand, tumor recurrence is not predictable, even in the case of unfavorable tumor biology, and the shortage of organs available for transplantation suggests alternative policies.

For these reasons, in the present study we chose to investigate the role of miRNAs in tumor recurrence after surgery in a selected series of “histologically advanced” HCC, i.e., HCC with high Edmondson grade (3 or 4), and/or presence of MVI. In the first testing set we selected two retrospective groups of recurrent and non-recurrent patients (groups A and B), all characterized by unfavorable histopathological features; we then recruited a prospective validation set (group C) with the same clinical characteristics. Previous studies found a correlation between miRNA expression and a higher risk of recurrence after liver resection [24–27] or liver transplantation [28, 29], but to the best of our knowledge none of these studies focused on a population with aggressive histological features (micro-vascular tumor invasion and/or poor differentiation degree), and therefore with an *a priori* high expected recurrence rate.

From the miRNA card analysis and the subsequent validation with qRT-PCR, two promising miRNAs emerged, with a significant difference in expression between group A (free from recurrence at 5 years) and group B (recurrence after resection): miR-483-3p and miR-548e. miR-483-3p is known for its anti-apoptotic function and it was previously associated with HCC recurrence in resected HCC in patients within the Milan criteria [24]. miR-483-3p expression is heterogeneous across human malignancies, acting as tumorigenic or a tumor-suppressor according to the target [22, 30]. In a previous study by our Institution, we noticed that miRNA 483-3p displayed a differential expression in HCC with peculiar vascular modifications characterizing advanced HCC [31]. Our current results showed that miR-483-3p is up-regulated in the non-recurrent group of patients and downregulated in the recurrence group, thus acting as a tumor-suppressor, at least in HCC. The validation on the prospective group C showed that with a miR-483-3p up-regulation (mean fold increase 5.94) no HCC recurrences were recorded; conversely, with a miR-483-3p down-regulation (mean fold increase 0.21) 44% of cases experienced local recurrence after surgery. Of note, we chose to enroll both resected and transplanted patients in group C, in order to evaluate the feasibility of the miRNA study on both models: as a result, tissue miR-483-3p expression seems to be applicable in both models. However, a prospective validation on larger series of resected and transplanted patients is mandatory to reinforce these data. In order to assess the deregulated expression of miR-483-3p, we chose a pool of normal liver tissues to avoid confounding factors related with the heterogeneous expression of cirrhotic tissue. In addition, our series of HCCs is represented by histologically aggressive cases, in order to identify a putative biomarker to be used in this specific setting.

In order to try to elucidate the possible mechanisms behind the heterogeneous expression of miR-483-3p in HCC, we studied the IHC expression of IGF2, a widely recognized up-regulated gene in HCC [34]. The IGF2 locus harbors the mir-483 locus within its second intron, and indeed the upregulation of miR-483-3p has been related to IGF2 expression [22]. Our preliminary data confirm a co-regulation of IGF2 and miR-483-3p in neoplastic hepatocytes. Previous studies identified the loss of imprinting at the IGF2 locus as a cause of enhanced IGF2 expression in Wilms’ tumor [35]. Being co-expressed in the majority of cases, we can hypothesize that miR-483-3p and IGF2 might be activated by the same mechanisms, although dedicated studies are needed to confirm this hypothesis.

From a biological point of view, this study is affected by several limitations. First, the small number of cases in both the testing and the validation sets means that further confirmation in larger prospective cohorts is required. Second, the up-regulation of miR-483-3p was assessed after comparison with a pool of normal liver tissues. The actual meaning of “normal liver” is somewhat indefinite due the incidence of chronic liver diseases in the population that might represent the only reason for miR-483-3p deregulation in our series of patients apart from HCC. Finally, the mechanistic association between miR-483-3p and IGF-2 expression in HCC is beyond the scope of this study and should be investigated by *in vitro* dedicated studies. If our data are confirmed by others, miR-483-3p would become one of the few miRNAs - the first in HCC- with actual biomarker validation.

In spite of the high number of targets described, there is a lack of literature concerning the role of miR-548e in HCC. Here, for the first time, we report a possible role of miR-548e downregulation in HCC recurrence, with a tumor-suppressor function (like miR-483-3p). This is in line with the observation from Shi et al. that the overexpression of miR-548e inhibits cell proliferation and promotes apoptosis in human breast cancer cell lines [32]. Unfortunately, the recurrence-free survival curve in relation to miR-548e expression was not significant as for miR-483-3p: further prospective studies are needed to validate the expression of miR-548e as a single predictive variable.

In conclusion, the correlation between miRNAs and HCC recurrence reported by our data could help to select a different treatment algorithm in this group of patients: liver resection and follow-up or liver resection and liver transplantation. miRNA expression could be evaluated prior to surgery from a needle liver biopsy, and this evaluation could be applied before liver resection to decide the most appropriate surgical treatment (liver resection *versus* liver transplantation). The routine *in situ* analysis of a few miRNAs is likely to be a feasible approach, with no particular limitations─our technique being tested on paraffin-embedded tissue as well─ and no contraindications. The possibility in the future of achieving a serum miRNA profiling of patients makes miRNA study even more motivating.

The issue of HCC recurrence after liver transplantation and liver resection has a great impact on the Health System in terms of biological and economic cost. The relatively high rate of disease relapse clearly has consequences in the transplantation outcome as well, thwarting its benefits. Early identification of those patients with higher risk of tumor recurrence is likely to have an important impact on both patient survival and on the number of successful transplantations, since the organs not allocated for these high-risk patients could be donated to other candidates. In a *scenario* where the discrepancy between the available organs and the candidates for transplantation is a major issue, a better policy for organ allocation seems to be a very important investment for the health system.

## MATERIALS AND METHODS

### Case selection: patients and histopathological features

The present tissue study was approved in advance by the Ethics Committee of the S.Orsola-Malpighi University Hospital (protocol code CTP-13-01, reference number 228/2013/O/Tess). Patients were treated according to the ethics guidelines of the 1975 Declaration of Helsinki (6^th^ revision, 2008). Written informed consent was obtained from each patient at the time of surgery.

For the purposes of the present study, all the selected tumors were “histologically advanced”, defined by Edmondson grade 3 or 4, and/or presence of microvascular invasion. Other collected histopathological features were: infiltrative margins, solid architecture, and tumor size.

Finally, 54 HCC patients were selected and assigned to 3 groups:

- “group A”: 15 retrospective surgically resected patients, with no HCC recurrence in 5 years of follow-up; patients were 11 males and 4 females, mean age 63.00±12.95 years; mean follow-up 2618.2±405.9 days.
- “group B”: 19 retrospective surgically resected patients with HCC recurrence within 1 year of follow-up; patients were 17 males and 2 females, mean age 62.11±8.59 years; mean follow-up 671.56±527.62 days.
- “group C”: 20 prospective resected patients (10 resected and 10 transplanted patients). Patients were 17 males and 3 females, mean age 60.25±15.05. Mean follow-up was 828.25±403.17 days, with 4 recurrence cases recorded.

Patients of groups A and B were retrospectively enrolled from January 2009 to June 2014 and patients of group C were prospectively enrolled from September 2013 to January 2015. Cirrhosis was present in 40 (74%) patients. The primary etiology of the underlying liver disease was: HCV for 25 (46.3%) patients, HBV for 14 (25.9%), biliary disease for 2 (3.7%), alcohol for 4 (7.4%) and metabolic syndrome for 3 (5.6%); only 2 cases (3.7%) were reported as cryptogenic cirrhosis. In 4 (7.4%) cases, HCC evolved from livers without patent disease. Before surgery, 19/54 patients were subjected to local ablative therapy: percutaneous ethanol injection (3 patients, 5.5%), radiofrequency ablation (7 patients, 13%), transarterial chemoembolization (13 patients, 24%), or transarterial radioembolization (2 patients, 3.7%). The locally treated nodules were obviously different from the nodules analyzed in the present study, since necrotic nodules were excluded from the miRNA expression analyses.

Baseline data on patient and cancer characteristics are listed in Tables 1 and 2, respectively.

All tissue samples, promptly fixed in formaldehyde 4% directly in the operating room and buffered pH 6.9 before routine processing, were routinely processed, diagnosed as HCC, and enrolled on the basis of the “histologically advanced” features by two different pathologists (FV and AD). The same two pathologists carefully evaluated histological sections prior to RNA extraction in order to select tissue block with at least 70% enrichment in neoplastic cells over the total cell population.

### miRNA arrays

#### RNA extraction

RNA was extracted from formalin-fixed and paraffin-embedded sections by a commercial kit (FFPE Recover All, Life Technologies, Carlsbad, CA, USA). After dewaxing with xylene, tissue was recovered from the slides by adding 10 μl of digestion buffer, following the manufacturer’s instructions. The RNA quality and concentration were evaluated by using a ND-1000 spectrophotometer (NanoDrop, Fisher Thermo, USA); RNA was considered pure if A260/A280 ratio was between 1.9 and 2.1.

#### Megaplex reverse transcription reaction and cDNA pre-amplification

The reverse transcription assay was performed using the TaqMan^®^ MicroRNA Reverse Transcription Kit associated with the Megaplex RT Primers Pool A and B (Life Technologies). Samples were loaded in the GeneAmp PCR System 9700 (Life Technologies) following the manufacturer’s reaction conditions. A second step of pre-amplification was performed by means of the TaqMan^®^ PreAmp Master Mix associated with Megaplex PreAmp Primers Pool A and B (Life Technologies), in order to pre-amplify multiple cDNAs. Samples were loaded in the GeneAmp PCR System 9700 following the manufacturer’s reaction conditions. The pre-Amp products were diluted in 75 μl of H_2_O.

### RT-PCR arrays

Relative quantitation of targets was performed with TaqMan Human microRNA Arrays 384-wells (Gene expression Micro Fluidic card, Array A v2.1 and Array B v3.0, Life Technologies). PCR Reaction mix consisted of 450 μl of TaqMan Universal Master Mix II no Amperase UNG (Life Technologies), 9 μl of diluted Pre-Amp product with primer Pool A or B and 441 nuclease-free water. PCR reaction mix (100 μl) was dispensed in each fill port of the 384 plate in the respective arrays. Amplification was performed on the Real Time 7900HT System (Life Technologies) and data were collected with the SDS software v2.2 (Life Technologies). Cycling conditions were as follows: 2 min at 50°C, 10 min at 94.5°C, 30 s at 97°C (40 cycles), 1 min at 59.7°C. The transcription levels were normalized using the global normalization method [33]. The expression values for HCC are presented as fold expression in relation to controls; the actual values were calculated using the 2−ΔΔCT equation, where ΔΔCT = [CT Target − CT Rnu44](target sample) − [CT Target − CTRnu44] (control sample). The control used was represented by a pool composed of 10 healthy livers obtained from virus-free and cancer-free multiorgan donors [31].

### Quantitative real-time polymerase chain reaction (qRT-PCR) validation for miR-483-3p and miR-548(e)

#### Multiplex reverse transcription reaction and cDNA pre-amplification

miR-483-3p and miR-548(e) were reverse-transcribed using the TaqMan^®^ MicroRNA RT kit and the associated miRNA-specific stem-loop primers. According to the manufacturer’s indications (MicroRNA Assay 5X, Life Technologies, Carlsbad, CA, USA), we created a custom pool consisting of 10 μl of each primer (miR-548(e), 002881 and miR-483-3p, 002339) and the reference gene (RNU-44, 001094), in a total volume of 1000 μl with nuclease-free H_2_O. The RT reaction mix had a final volume of 7.5 μl and contained 3 μl of custom pool 5X, 1.5 μl of RNA, 0.5 μl of Nuclease-free water, 0.15 μl of dNTP, 1.5 μl of MultiScribe™ Reverse Transcriptase (50 U/μL), 0.75 μl of RT buffer, 0. 1 μl RNase inhibitor. Samples were loaded in the GeneAmp PCR System 9700 following the manufacturer’s reaction conditions. The pre-amplification step was also performed with a custom pool of primers (TaqMan^®^ MicroRNA Assay 20X, Life Technologies, Carlsbad, CA, USA) with specific probes for miR-548(e), miR-483-3p and RNU-44. The preamplification reaction had a final volume of 20 μl and contained 3 μl of custom pool 20X, 2 μl of cDNA, 5 μl of Nuclease-free water, 10 μl of TaqMan^®^ PreAmp Master Mix. Samples were loaded in the GeneAmp PCR System 9700 following the manufacturer’s reaction conditions.

#### qRT-PCR

miRNA quantitation was carried out in duplicate by means of the TaqMan^®^ Universal Master Mix II No AmpErase UNG kit (Life Technologies, Carlsbad, CA, USA). The PCR reaction mix had a final volume of 10 μl and contained 5 μl of TaqMan^®^ Master Mix, 0. 5 μl of TaqMan^®^ fluorescent probes specific for each miRNA (20X TaqMan MicroRNA Assay, Life Technologies, Carlsbad, CA, USA), 2.5 μl of water, 2 μl of cDNA. Fluorescent signal was detected with Real Time 7900HT System and analyzed with the SDS v2.2 software. The transcription levels were normalized using RNU-44 as reference gene. The expression values for recurrent and non-recurrent HCC are presented as fold-increase in relation to control. The actual values were calculated with the 2−ΔΔCT equation, where ΔΔCT = [CT_Target_ – CT_RNU44_] (target sample) − [CT_Target_ – CT_RNU44_] (control sample).

### Immunohistochemistry

Immunohistochemistry (IHC) for IGF2 (anti-IGF-II, clone S1F2, mouse monoclonal IgG_1_, Upstate Biotechnology) was manually performed on formalin-fixed and paraffin-embedded HCC tissue from cases of group C (prospective group) with NovoLink Polymer Detection Kit (Novocastra, Newcastle, UK), as previously described [36]. Briefly, antigen retrieval was performed with citrate buffer pH 6, heat mediated, and sections were incubated at room temperature for 1hour with IGF-II diluted at 1:200. Cell nuclei were counterstained with Mayer’s hematoxylin.

### Statistical analysis

Statistical analysis was performed by means of SPSS software for Windows, V.20, and Prism 5 software (GraphPad Software). Variables were expressed as means ± standard deviations, ranges, and frequencies. Receiver Operator Characteristics (ROC) curve was used to assess the best cut-off for miRNA expression in relation to tumor recurrence; the Kaplan-Meier univariate analysis was applied to study RFS in patients with different miRNA cut-offs.

MicroRNA Arrays results were analyzed with ExpressionSuite Software v1.0 (Life Technologies). First, Ct values were normalized with the global normalization method and then normalized with the control groups (pool of 10 healthy livers). The Student’s t test compared the means of the normalized Ct values between two groups. The p values obtained for arrays were adjusted to control the type I error rate using the Benjamini–Hochberg method [37]. miRNAs with a significant differential expression compared to controls (in arrays) were plotted on a Volcano plot. The qRT-PCR data for single assays to evaluate miRNA 483-3p and 548e expression were analyzed assuming the null hypothesis that the cycle threshold (CT) differences between target and reference genes will be the same in HCC group versus the control pool [38]. The continuous variables of qRT-PCR were analyzed with the Wilcoxon-matched pairs test (comparison between ΔΔCT of control pool and each HCC group). ANOVA test was used to study the correlation between IHC and qRT-PCR results. Statistically significant refers to P<0.05 and statistically highly significant to P<0.001.

## SUPPLEMENTARY MATERIALS TABLE




