# Supplementary material for: Tissue miRNA 483-3p expression predicts tumor recurrence after surgical resection in histologically advanced hepatocellular carcinomas
Source: Oncotarget. 2018 Apr 3;9(25):17895–905. doi: 10.18632/oncotarget.24860 (PMC5915163; doi:10.18632/oncotarget.24860)
Supplement: Supplementary file 1 [file oncotarget-09-17895-s001.pdf]

Tissue miRNA 483-3p expression predicts tumor recurrence after surgical resection in histologically advanced hepatocellular carcinomas

SUPPLEMENTARY MATERIALS

Supplementary Table 1: Significant miRNA expression levels in recurrent and non-recurrent HCCs. The table lists the raw data used to calculate the miRNA expression levels in recurrent and non-recurrent HCC. Final values are expressed as fold-increase (RQ) and obtained with the $2^{-\Delta\Delta CT}$ equation. Only significant RQ differences are listed.

See Supplementary File 1
